# Commentary: Echolocation and vision complement each other in two bat species

**DOI:** 10.3389/fphys.2015.00236

**Published:** 2015-08-24

**Authors:** Mariana L. Melcón

**Affiliations:** Fundación CethusOlivos, Argentina

**Keywords:** echolocation, evolution, bimodal sensing, bats, navigation

Many nocturnal mammals have developed senses such as vision, olfaction, and whisking to orient, navigate and forage in dark environments. Most bats, however, have evolved developing echolocation—an active sense that works as a sonar system. While it is clear the advantages that echolocation possess in complete darkness, it is not well understood why bats count on echolocation and nocturnal vision to operate at light levels as encountered at dusk.

In the research article “It's not black or white–on the range of vision and echolocation in echolocating bats,” Boonman and colleagues were able to shed light into the probable use of two complementary sensory systems used by echolocating bats. For this, they used both a theoretical as well as an empirical approach to compare the performance of vision and echolocation in two bat species whose peak activity takes place immediately after sunset—i.e., intermediate light levels. Both methods used in the manuscript conveyed similar conclusions: vision performed better at detecting large objects such as trees, whereas echolocation worked better in the detection of small objects, disregarding light levels (Boonman et al., 2013).

By using *Rhinopoma microphyllum* and *Pipistrellus kuhlii*, Boonman et al. (2013) ensured to include two bat species with very different echolocation signals that exploit two different habitats: *R. microphyllum* is an open space aerial hawker which hunts on large insects, and *P. kuhlii* is an edge space aerial hawker which specializes in catching small insects. Authors found for both experimental models that echolocation is superior for navigation under extreme darkness and for detecting and tracking small objects, such as insects. For example, they estimated for *P. kuhlii* an acoustic detection range for a mosquito of 2–4 m, and visual detection range of 0.5–1 m, depending on the method used. Similarly, *R. microphyllum*'s detection range was estimated at 2–5.5 m with echolocation, while only 0.5–1.5 m with vision.

What are the advantages of echolocation over vision? Echolocation happens to work better for continuous tracking of objects since it is independent on the contrast. It also provides animals with a more accurate estimation of distance to the target, speed, and distance to the background. So why not just use echolocation? Vision has its advantages as well, such as not suffering from interference (proceeding from the vocalizations of conspecifics), and granting animals a larger detection range when perceiving a large object (because light attenuates slower than sound). Note that the detection of large objects in vision refers exclusively to bats, who have a somewhat more limited vision than most diurnal mammals or birds.

While echolocation sounds like a very nice sense to have, what would have been the adaptive advantage of acquiring such a unique sensory system, if most nocturnal animals have survived for millions of years without the need of a sonar system? In 1998, Simmons and Geisler suggested that bats evolved by first navigating and detecting obstacles by means of vision, while passively listening for prey-generated sounds, then they started using echolocation and vision for orientation. They next used echolocation for tracking flying prey, followed by prey detection and tracking using echolocation, and finally exclusive reliance on echolocation for prey detection and tracking while orientation and obstacle detection were in charge of vision (Simmons and Geisler, 1998). Assuming this evolutionary scenario, echolocation could adapt gradually for a better detection of increasingly small targets in parallel to using vision for orientation and navigation. Even if the detection range of echolocation were similar to vision (and not necessarily superior), the selective advantage of the echolocation evolution is still valid since the integration of information proceeding from multiple sensory systems (in this case, vision and echolocation) leads to a single yet probably more accurate image of their surroundings (Figure 1).

**Figure 1:**
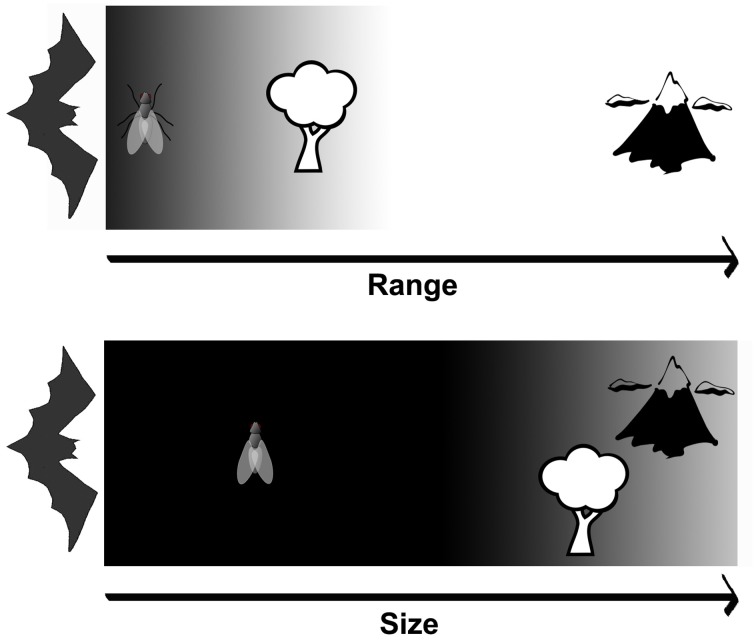
**Detection scenario for echolocating bats at intermediate light levels**. When it comes to range, the upper panel shows that both echolocation and vision (gray background) may be providing complementary information about the short distance environment, whereas only vision (white background) contributes for long distances. Inversely, the lower panel depicts that while large objects can be successfully detected by both sensory modalities (gray background), small objects can be only detected and tracked by echolocation (black background) at dusk.

It is clear from the literature and the evidence that Boonman et al. (2013) provide that echolocation is capable of detecting small targets. In fact, echolocation has evolved allowing bats to specialize in alternative detection modes: some animals can detect flutter by using Doppler shifts, others can glean prey from vegetation. The different specializations of echolocation might have caused radiation of bats into both different climatic zones and niches.

Even though the authors acknowledge to have disregarded the costs associated to the evolution of echolocation, the advantage of acquiring such a specialized sensory system is likely to pay off: From the emerging aquatic biomass, small chironomids (family of the mosquitoes, defined in the article as smaller than 7 mm) constitute 48–85%. Their activity peak takes place at dusk, and in many cases they are available all year round. The significant biomass and extended availability of this niche would seem like a loss to be almost untapped if it were not for echolocation.

In conclusion, for echolocating bats sensing the environment does not seem to be black nor white. Using a combination of echolocation and vision allows them to benefit from the advantages of both sensory systems. The integrated images of both senses have enabled them to have a more accurate mental representation of their surroundings with a larger dynamic range in multiple dimensions (such as size of objects detected, distances, and even temporal resolution). This acquisition allowed them to conquer an almost unexploited yet significant niche.

## Conflict of interest statement

The author declares that the research was conducted in the absence of any commercial or financial relationships that could be construed as a potential conflict of interest.
